# The effect of carbodiimide on push-out bond strength of fiber posts and endogenous enzymatic activity

**DOI:** 10.1186/s12903-023-03067-y

**Published:** 2023-06-16

**Authors:** Uros Josic, Claudia Mazzitelli, Tatjana Maravic, Allegra Comba, Milena Cadenaro, Ivana Radovic, Maicon Sebold, Gianluca Turco, Lorenzo Breschi, Annalisa Mazzoni

**Affiliations:** 1 Department for Biomedical and Neuromotor Sciences, Dental Clinic, DIBINEM, University of Bologna, Via San Vitale 59, Bologna, 40125 Italy; 2 Department of Surgical Sciences, University of Turin, Turin, Italy; 3 Department of Medical Sciences, University of Trieste, Trieste, Italy; 4 Institute for Maternal and Child Health–IRCCS “Burlo Garofolo,”, Trieste, Italy; 5 Clinic for Paediatric and Preventive Dentistry, School of Dental Medicine, University of Belgrade, Belgrade, Serbia; 6 Department of Restorative Dentistry, Operative Dentistry Division, Piracicaba Dental School, University of Campinas, Piracicaba, SP Brazil

**Keywords:** Carbodiimide, Radicular dentin, Push-out bond strength, MMPs, In situ zymography

## Abstract

**Background:**

To investigate the effect of 0.3 M 1-ethyl-3(3-dimethylaminopropyl) carbodiimide (EDC) aqueous solution pretreatment on push-out bond strength (PBS) and matrix-metalloproteinases (MMPs) activity within radicular dentin when different post cementation strategies were employed.

**Methods:**

One hundred and twenty monoradicular human teeth were endodontically treated and randomly divided into six groups, depending on the cementation strategy and root dentin pretreatment (n = 20): EAR: cementation with an etch-and-rinse adhesive (LuxaBond Total Etch, DMG) and resin cement (LuxaCore Z Dual, DMG); EAR/EDC: 1 min EDC pretreatment after etching + EAR; SE: cementation with a self-etch primer (Multilink Primer, Ivoclar Vivadent) and corresponding cement (Multilink Automix, Ivoclar Vivadent); SE/EDC: self-etch primer + EDC pretreatment + SE; SA: cementation with a universal self-adhesive cement (RelyX Universal, 3 M); SA/EDC: EDC pretreatment + SA. Slices were submitted to PBS test and interfacial nanoleakage evaluation 24 h after cementation or after thermocycling (40.000 cycles, 5–55 °C). To investigate the effect of EDC on MMPs activity, 4 additional first maxillary premolars per group were processed for in situ zymography analysis. Multivariate ANOVA and post hoc Tukey tests were used to analyze PBS values. The data from in situ zymography were analyzed with Kruskal-Wallis test and Dunn’s pairwise multiple comparison procedures (α = 0.05).

**Results:**

The variables “EDC pretreatment”, “root region” and “thermocycling” significantly influenced PBS (p < 0.05), while the variable “cementation strategy” had no influence (p > 0.05). Thermocycling significantly reduced PBS in SE and SA groups (p < 0.05). EDC was effective in preserving PBS after artificial aging. EDC pretreatment significantly reduced enzymatic activity at baseline in EAR and SE groups, and in SA group after thermocycling (p < 0.05).

**Conclusions:**

The use of EDC prevents the reduction of bond-strength values after artificial aging and silences endogenous enzymatic activity within radicular dentin when different cementation strategies were employed.

## Background

Resin luting agents have become the current material of choice for fiber post cementation. Posts can be cemented through dual-cure resin cements, requiring the application of etch-and-rinse (EAR) or self-etch (SE) adhesive systems, or by means of self-adhesive (SA) cements that do not involve a previous dentin pretreatment with an adhesive [1]. At least two separate clinical steps are needed when cementing posts with an adhesive system, and therefore these materials are referred to as multi-step resin cements [2]. Application of multi-step resin cements into the root canal leads to the formation of the hybrid layer (HL) – a structure that is composed of demineralized collagen matrix reinforced by resin tags [3–6]. On the other hand, SA luting resins do not form a classic HL, but can chemically interact with dentin due to the presence of acidic functional monomers in their composition [3, 7]. The thickness of the interaction zone between dentin and SA cements does not exceed 1 μm, and contains less tag-like structures compared to multi-step resin cements [8].

Although the exact mechanism for the retention of fiber posts inside root canals has not been entirely clarified [9], the integrity of the HL/interdiffusion zone is considered one of the factors that might play an important role in preventing post dislodgment [10]. The degradation of the resin-dentin interface is likely to occur due to the presence of endogenous enzymes found in both coronal and radicular dentin [11–14]. The most widely investigated endogenous enzymes are matrix-metalloproteinases (MMPs), which are trapped and inactive within mineralized dentin after tooth development. However, these enzymes become active during adhesive procedures and can degrade poorly infiltrated collagen fibers, eventually leading to the loss of bond-strength of resin-dentin restorations [15, 16]. The presence of zinc and calcium ions is essential for the MMP activity, since they play an important role in the activation and preservation of the tertiary structure of the enzyme, respectively [5].

One of the strategies to prevent the reduction of bond-strength caused by MMP activity is the inhibition of such enzymes coupled with the increase of the collagen resistance to degradation. For this purpose, the application of a cross-linking agent, 1-ethyl-3-(3-dimethylamino-propyl) carbodiimide (EDC), on demineralized dentin was proposed [15]. Indeed, promising results in preserving bond-strength values and inhibiting MMP activity were reported when EDC was applied on coronal dentin during adhesive procedures [17–20]. EDC was shown to preserve the bond-strength values, cross-link the collagen and silence MMP activity within coronal dentin, even after 5 years of artificial aging, when EAR or SE adhesives were used during adhesive protocols [21]. However, only few studies investigated the effect of EDC on the push-out bond strength of fiber posts [22–25]. Furthermore, as far as the authors of this study know, no study evaluating the effect of EDC on endogenous enzymatic activity comparing all 3 fiber post cementation strategies is available in the literature. Therefore, this study aimed to investigate the effect of EDC on the bond-strength of adhesively luted fiber posts, as well as on the enzymatic activity within root dentin when 3 cementation strategies were used (EAR, SE, and SA). The following null hypotheses were tested: (1) EDC pretreatment would not influence push-out bond strength values of adhesively luted fiber posts at baseline or artificial aging; (2) EDC pretreatment would have no effect on enzymatic activity within radicular dentin at baseline or after artificial aging.

## Materials and methods

### Endodontic treatment procedures

One hundred and twenty freshly extracted, intact, monoradicular human teeth (central maxillary incisors and first mandibular premolars) [26] were kept in 0.5% chloramine solution at 4^o^C for no longer than 2 months [27] after extraction. The study protocol was approved by the Ethics Committee of the Department for Biomedical and Neuromotor Sciences (DIBINEM), University of Bologna, Italy (protocol N°: 71/2019/OSS/AUSLBO). In order to obtain a standardized root length of 14 mm, each tooth was sectioned below the enamel-cementum junction using a low-speed diamond saw (Microremet, Remet, Casalecchio di Reno, Italy), under water cooling, perpendicular to the long axis of the tooth. Root canal preparation was performed with Pathfiles (#1-2-3) and ProTaper (S1-S2-F1-F2-F3) (Dentsply Sirona, York, PA, USA) until the instruments reached the working length (1 mm from the apical foramen). During canal shaping, irrigation was performed with 5% sodium hypochlorite (NaOCl) solution (Niclor 5; Ogna, Muggiò, Italy) between different instruments. The final rinse was performed with 17% ethylenediaminetetraacetic (EDTA) acid for 1 min and 5% NaOCL, after which the canals were dried using paper points. According to the continuous wave technique, the canals were filled with endodontic sealer (AH-Plus, Dentsply Sirona, Konstanz, Germany), medium-sized gutta-percha points with DownPack (Hu-Friedy, Chicago, IL, USA) and warm gutta-percha (Obtura III, Analytic Technologies, Redmond, WA, USA). The canal entrance was then temporarily sealed with a glass-ionomer cement (Fuji VII, GC Corp., Tokyo, Japan) and the samples were stored in Eppendorf tubes for the next 24 h at 37 °C and 100% relative humidity.

### Fiber post cementation

After removing the temporary coronal sealing, the gutta-percha was removed with a size 3 Gates-Glidden bur, and a post-space preparation of 8 mm was carried out using a slow dental handpiece and fiber post drill (RelyX fiber post drill, Size 2, 3 M, Neuss, Germany). The drills were replaced after 10 applications. The prepared root canals were irrigated with 5 ml of distilled water and dried with paper points. Before luting procedures, a size 2 fiber post (RelyX™, 3 M, Neuss, Germany) was inserted into the canal to check if it reached the working length, after which the coronal part of the fiber post outside the canal was cut with a diamond bur. The teeth were then randomly assigned, by toss of a coin, to one of the following groups, according to luting agent and dentin pretreatment protocol (n = 20):

EAR group: dentin etching with 37% phosphoric acid for 15 s (Etching Gel, DMG, Hamburg, Germany), abundant water rinsing for 30 s, and root canal drying with paper points. Application of Pre-Bond (DMG) for 15 s, drying with paper points, followed by application of previously prepared Bond A/B (DMG). Gentle air drying and fiber post cementation with Luxacore Z Dual (DMG);

EAR/EDC group: the same cementation protocol as in the EAR group was used, with 0.3 M EDC-containing aqueous primer application for 1 min, followed by drying with paper points, immediately after the acid-etching step;

SE group: dentin was treated with Multilink Primer (Ivoclar Vivadent, Schaan, Liechtenstein) for 30 s and dried with paper points, followed by fiber post cementation with Multilink Automix/Multilink Primer (Ivoclar Vivadent);

SE/EDC group: dentin was treated with Multilink Primer for 30 s, dried with paper points, and treated with 0.3 M EDC-containing aqueous primer for 1 min. Root canals were dried with absorbent paper points, a layer of Multilink Primer was applied for 30 s, and the fiber posts were cemented with Multilink Automix (Ivoclar Vivadent);

SA group: fiber post cementation with RelyX Universal (3 M); SA group: cementation with RelyX Universal (3 M);

SA/EDC group: dentin pretreatment with 0.3 M EDC-containing aqueous primer for 1 min, drying with absorbent paper points, followed by fiber post cementation with RelyX Universal (3 M).

The details on fiber post surface treatment, chemical compositions and application modes of the cements used in the study are shown in Table 1. Light curing was performed immediately after FRC post insertion by placing the light source (1470 mW/cm2, Elipar Deep cure, 3 M) in close contact to the root canal entrance for 40 s. Endodontic treatment, obturation, post space preparation and cementation were performed by one single experienced operator (U.J.). In order to prevent dehydration, the teeth were stored at 37 °C and 100% humidity for 24 h. Half of the teeth from each group were allocated to the baseline subgroup (T_0_, randomization performed by toss of a coin), while the other half was assigned to artificial aging subgroup (T_t_) Subsequently, the roots were embedded in epoxy resin and perpendicularly sectioned in 1-mm-thick sections using a low-speed diamond saw (Microremet, Remet, Bologna, Italy). The coronal side of each slice was signed with an indelible marker to later ensure the exact positioning during the push-out bond strength test. Two slices from the apical, middle and coronal thirds of the root were obtained from each tooth. In the slices from T_0_, the push-out bond strength test was performed 24 h after cementation, while a thermocycling protocol was employed for the T_t_ specimens (40.000 cycles in a thermocycling machine (SD Mechatronik Thermocycler THE-1100 / THE-1200, Mechatronik, Germany), 5–55 ^o^C, dwell time in hot/cold water 30 s, dry time 3 s). This protocol was in accordance with a previous study [28] and fulfilled the requirements given by the Academy of Dental Materials guidance on in vitro testing [27].

**Table 1:** The details of fiber post surface pretreatments, chemical compositions, batch numbers and application modes of the cements

Resin cement	Composition	Application mode	FRC post preparation
Luxacore Z Dual, DMG (LOT 207,905)	Bis-GMA, UDMA, Barium glass, colloidal silica, nanocomposite, zirconium dioxide 71% weight	Apply DMG etching gel for 15 s on radicular dentin, rinse with water for 15 s. Dry the canal with paper points. Work 1 drop of prebond (Luxacore Total Etch) to dentin for 15 s, remove the access with paper point, gently air-dry. Mix Bond A and Bond B (1:1) and apply to dentin surface for 20 s using a microbrush, gently air-dry. Dispense the cement in the post space and insert the post.	Clean with alcohol and air-dry for 5 s. Apply a layer of silane coupling agent (Ultradent) for 60 s and gently air-dry.
Multilink Automix, Ivoclar Vivadent (LOT Y481141)	Dimethacrylate and HEMA, barium glass and silica filler, ytterbiumtrifluoride (68 wt %), catalysts, stabilizers, pigments	Mix Multilink Primer (1:1) and apply with an endobrush to radicular dentin for 30 s. Remove the access with an absorbent paper point. Dispense the cement in the post space and insert the post.	Clean with alcohol and air-dry for 5 s. Apply a layer of Monobond Plus (Ivoclar Vivadent) for 60 s and gently air-dry.
RelyX Universal, 3 M (LOT 7,182,977)	BPA derivative free dimethacrylate monomers, phosphorylated dimethacrylate adhesion monomers, photoinitiator system, novel amphiphilic redox initiator system, radiopaque fillers and rheological additives, pigments	Dispense in the post space and insert the post.	Clean with alcohol and air-dry for 5 s.

### Push-out bond strength test (PBS)

A push-out bond strength test (n = 10) at baseline and after thermocycling was performed in order to investigate the resistance of fiber posts to dislodgment from the root canal space. The thickness of root slices was measured using a digital caliper (Starrett 727, Starrett, Itu, SP, Brazil) with ± 0.01 mm accuracy. The slices were then placed on 1mm^2^ graph paper and photographs were taken with a digital camera (D 7200, Nikon, Japan), after which the coronal and apical diameters of the posts were measured using the ImageJ software (National Institute of Health, Bethesda, MD, USA). The push-out test was performed using a universal testing machine (Instron 4465, Instron, Norwood, MA, USA) by applying an axial load force at a constant crosshead speed of 0.5 mm/min in the apical-coronal direction. The load at the moment of failure (manifested by the dislodgment of the post) was recorded in Newtons (N) and then converted to mega Pascals (MPa) by dividing the load (in N) by the bonded surface area (SL - in mm^2^). The following formula was used to calculate the bonded surface area:

$${\rm{SL = }}\left( {{\rm{\pi }}\left( {{\rm{R + r}}} \right)} \right){\rm{*}}{\left( {{{\left( {{{\rm{h}}^{\rm{2}}}{\rm{ + }}\left( {{\rm{R - r}}} \right)} \right)}^{\rm{2}}}} \right)^{{\rm{0}}{\rm{.5}}}}{\rm{,}}$$

where R was the coronal diameter of the canal with the post, r the apical diameter and h the thickness of the slice [23].

The fractured specimens were analyzed by one operator (C.M.), blinded to the groups, under a stereomicroscope at 40x magnification (Stemi 2000-C; Carl Zeiss Jena GmbH). Failure modes were classified as follows: adhesive failure between dentin and luting agent (AD), adhesive failure between luting agent and fiber post (AP), cohesive failure within the luting agent layer (CC), cohesive failure within the fiber post (CP), and mixed failure (M).

### In situ zymography analysis of the resin-dentin interfaces

The roots of freshly extracted maxillary first premolars (n = 4) were endodontically treated, after which fiber post cementation was performed as previously described for the push-out bond strength test. A split tooth design was employed to account for the variability of intrinsic dentin enzymatic activity among different teeth. One of the roots of each tooth (EDC-primed root) was treated with 0.3 M EDC solution for 1 min during luting procedures, whereas the other root (control) was not primed with the experimental agent. After 24 h hours of storage at 37^o^C in a humid chamber, 1 mm-thick slabs from the middle portion of the roots were obtained from the prepared specimens using a low-speed diamond saw (Microremet, Remet, Bologna, Italy) under water cooling. Half of the specimens was immediately processed for in situ zymography analysis, while the other half was subjected to artificial aging in a thermocycling machine (following the same protocol of the push-out bond strength test).

Each specimen was glued to a microscope slide, ground down to a thickness of approximately 50 μm, and polished. In situ zymography was performed following the previously reported protocol [20]. Self-quenched, fluorescein-conjugated gelatin mixture (E-12,055; Molecular Probes, Eugene, OR, USA) was placed on the specimens, which were then protected with a coverslip. Specimens were then incubated for 12 h at 37 °C in a humid, dark chamber with no direct water contact. A confocal laser scanning microscope (488 nm excitation wavelength; 530 nm emission wavelength; Model A1-R; Nikon, Tokyo, Japan) was used to observe the specimens. For each assembly, a series of images with a standardized rectangular selected area was made (one image at each µm into the depth of the sample) to show the hydrolysis of the quenched fluorescein-conjugated gelatin substrate, observed as green fluorescence. The ImageJ software (National Institutes of Health, Bethesda, MD, USA) was used to quantify the integrated density of the fluorescence signal, which corresponds indirectly to the endogenous enzymatic activity. All images were taken and analyzed by experienced investigators (T.M. and U.J., respectively) who were blinded to the groups.

### Interfacial nanoleakage (NL) expression

Additional teeth (n = 4) were used to quantify the interfacial NL expression. Endodontic treatment, fiber post cementation, and cutting procedures were carried out as previously described for the push-out bond strength test. The NL analysis was performed at baseline and after thermocycling. The specimens were prepared and covered with nail varnish, leaving a 1 mm-thick area of the bonding interface exposed, then they were immersed in a 50 wt% ammoniacal silver nitrate solution for 24 h. Following, specimens were photo-developed to reduce the diamine silver ions (Ag(NH_3_)_2_^+^) into metallic silver grains. Images of the adhesive interfaces were captured [20x magnification (Nikon E800 Nikon, Tokyo, Japan)], and the extent of interfacial NL was scored by a single observer (U.J.), unaware of the groups [29], using a four-point scale. Interfacial nanoleakage was scored based on the percentage of the adhesive surface showing silver nitrate deposition: 0 - no nanoleakage; 1 - nanoleakage of less than < 25%; 2 - nanoleakage between 25% and 50%; 3 - nanoleakage between 50% and 75%; and 4 - nanoleakage of more than 75% [30, 31].

### Specimen processing for scanning electron microscopy (SEM) analysis

After performing the push-out bond strength test, representative root slices from each group (N = 2) were selected and processed for scanning electron microscopy (SEM) analysis. The samples were prepared for SEM analysis following the previously described protocol [32], and the observations were performed using a scanning electron microscope (JSM 5200, JEOL, Tokyo, Japan) at 50x, 100x, and 500x magnification.

### Statistical analysis

The number of ten samples for each experimental group was determined by a preliminary power analysis, which was conducted to assure a statistical power of at least 90%, given the standard value of type I errors (0.05). The mean values used were 16 MPa for the control teeth and 18 MPa for the EDC-treated teeth, with within-group standard deviation of 3 MPa. These values were based on a previous publication [24]. The calculation was conducted via an online calculator available at www.dssresearch.com.

After checking the normality (Shapiro-Wilk test) and homoscedastic (modified Levene’s test) of the data, an analysis of variance (ANOVA) was performed to examine the effects of the dependent variables “EDC pretreatment”, “cementation strategy”, “root region”, and “thermocycling”, as well as the interaction of these factors on push-out bond strength. Pairwise comparisons were performed using Tukey’s post-hoc test. In addition, one-way ANOVA with the Bonferroni post-hoc correction test was conducted to evaluate differences among groups. Since the data obtained from the in situ zymography analysis were not normally distributed (Shapiro-Wilk test, p < 0.05), the Kruskal-Wallis test, followed by Dunn’s pairwise multiple comparisons, was run. All statistical analyses were conducted with the software Stata 12.0 (Stata Corp, College Station, Texas, USA) by a statistician blinded to the groups [29], and the significance was set for p < 0.05.

## Results

### Push-out bond strength

Bond strength data were expressed as means and standard deviations (in MPa) and are shown in Tables 2 and 3.

**Table 2:** Push-out bond strength values (MPa) with standard deviations after 24 h of artificial saliva storage. Different superscript upper letters indicate differences within the rows, different superscript lower letters indicate differences within the columns

	Control			EDC		
Groups	*Coronal*	*Middle*	*Apical*	*Coronal*	*Middle*	*Apical*
**EAR**	17.8 ± 3.3^Aa^	14.5 ± 2.3^Aa^	13.9 ± 3.2^Aa^	17.5 ± 4.8^Aa^	14.7 ± 4.9^Aa^	12.2 ± 4.3^Aa^
**SE**	18.8 ± 7.5^Aa^	15.8 ± 3.9^Aa^	12.9 ± 4.8^Aa^	18.0 ± 5.2^Aa^	13.6 ± 4.5^Aa^	12.0 ± 4.6^Aa^
**SA**	19.0 ± 5.6^Aa^	13.7 ± 3.3^ A,B a^	9.7 ± 3.2^B a^	20.5 ± 6.9^Aa^	12.6 ± 4^ A,B,a^	11.2 ± 4.3^Ba^

**Table 3:** Push-out bond strength values (MPa) with standard deviations after 40.000 thermocycles. Different superscript upper case letters indicate differences within the rows, different superscript lower case letters indicate differences within the columns

	Control			EDC		
Groups	*Coronal*	*Middle*	*Apical*	*Coronal*	*Middle*	*Apical*
**EAR**	14.0 ± 5.3^Aa^	10.8 ± 4.0^Aa^	9.1 ± 3.9^Aa^	17.4 ± 4.5^Aa^	14.1 ± 2.3^Aa^	15.5 ± 3.5^Aa^
**SE**	10.8 ± 4.1^Aa^	9.9 ± 4.1^Aa^	9.2 ± 3.5^Aa^	16.1 ± 8.5^Aa^	13.8 ± 5.3^Aa^	13.7 ± 3.9^Aa^
**SA**	11.6 ± 3.4^ A,Ba^	8.8 ± 3.0^Ba^	9.5 ± 1.6^Ba^	18.8 ± 4.^Aa^	11.6 ± 4^ A,Ba^	11.0 ± 3.4^ A,Ba^

Results of the factorial ANOVA showed the variables “EDC pretreatment” (p = 0.0001), “root region” (p = 0.0001), and “thermocycling” (p = 0.0001) significantly influenced the PBS values. The variable “cementation strategy” did not influence the push-out bond strength (p > 0.05). The interactions between “root region” and “thermocycling”, “EDC pretreatment” and “thermocycling” were significant (p = 0.0215 and 0.0001, respectively). There were no other statistically significant interactions (p > 0.05). EDC pretreatment was able to prevent the loss of bond-strength after thermocycling, regardless of the employed cementation strategy (Tables 2 and 3). Thermocycling significantly reduced PBS in the SE and SA control groups (P < 0.05) compared to the baseline values. Lastly, significantly lower PBS values were observed at baseline in the apical third in the SA control and EDC groups compared to the coronal portion of the root (p < 0.05).

### Failure mode

**Fig. 1:**
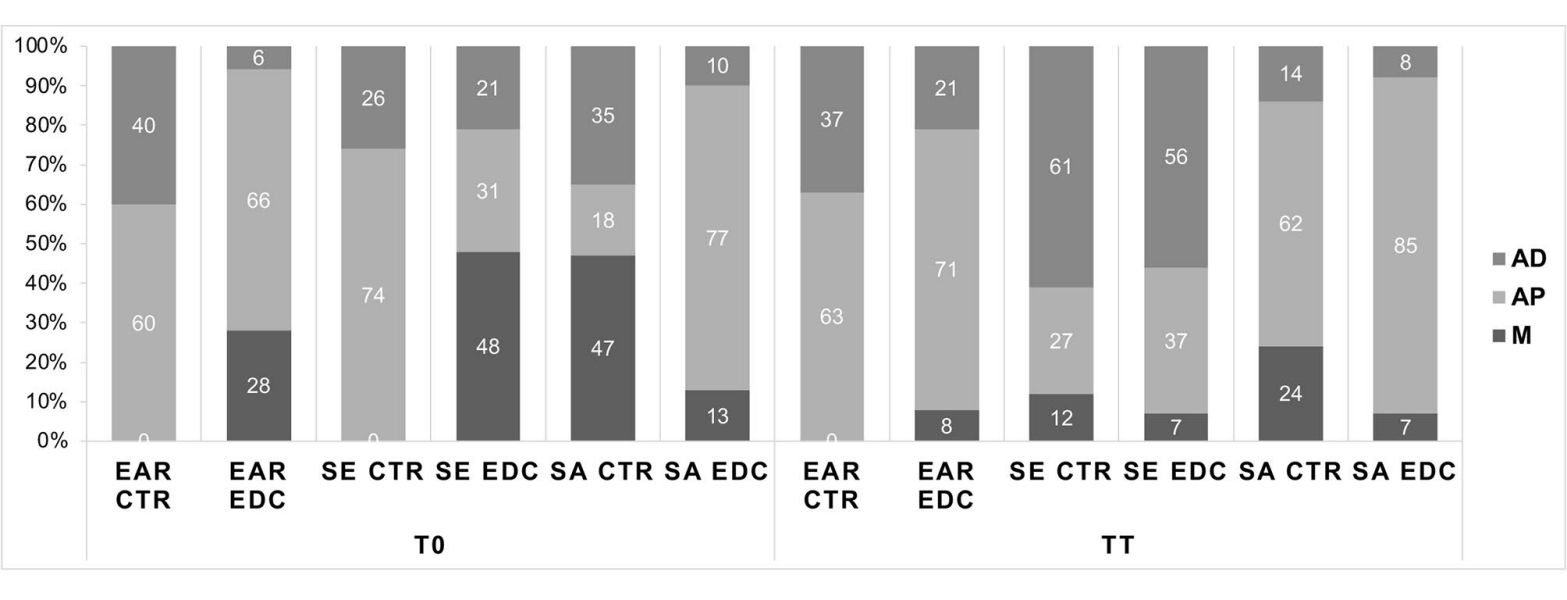
Failure mode of the dislodged specimens from three experimental groups. Data are expressed as percentages (%) of the total number of specimens tested for each group. Cohesive failures within the fiber post or failures within luting agent were not observed in any of the groups and were therefore not presented on the graph. Abbreviations: AD-adhesive failure between dentin and luting agent; AP-adhesive failure between luting agent and fiber post; M-mixed

Failure mode distribution of the tested specimens, expressed as percentages of the total number of tested slices, are summarized in Fig. 1. No cohesive failures within the fiber post nor failures within luting agent were observed in any of the groups. In general, artificial aging increased the percentage of adhesive failures between luting agent and dentin.

### In-situ zymography

**Fig. 2:**
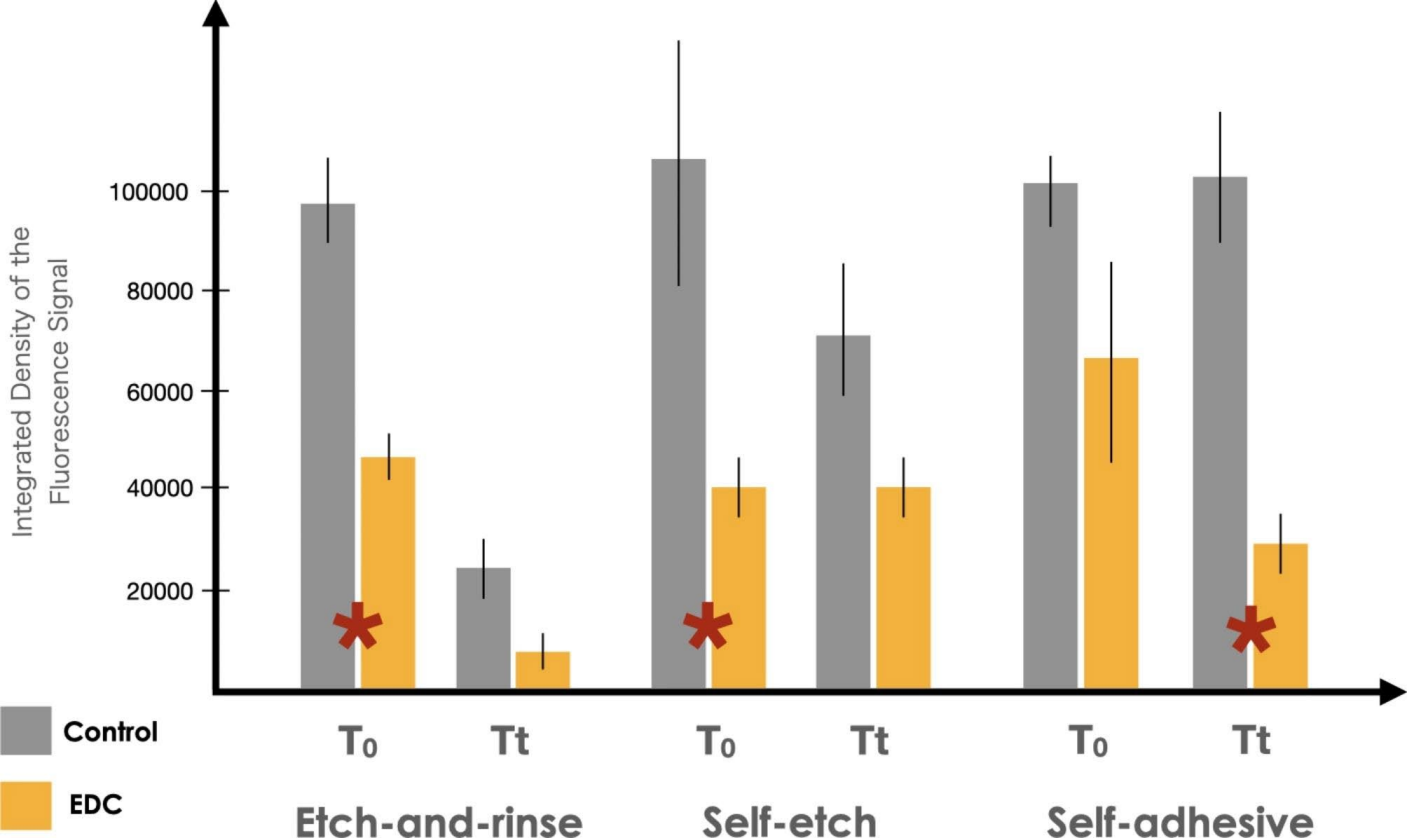
Gelatinolytic activity, expressed as the percentage of the green fluorescence within resin/dentin interfaces within radicular dentin created in experimental groups. Values are means and standard deviations. Red asterisk indicates statistically significant differences between the groups

The results obtained from the in situ zymography analysis of two-rooted premolar teeth are presented in Fig. 2. EDC pretreatment significantly reduced the enzymatic activity at baseline in the SE (p = 0.015) and EAR (p = 0.01) groups. Similarly, a significant difference was observed between control and EDC groups after thermocycling when the posts were bonded with SA cement (p < 0.001). Interestingly, thermocycling reduced the enzymatic activity in both control and experimental EAR groups (p < 0.05).

**Fig. 3:**
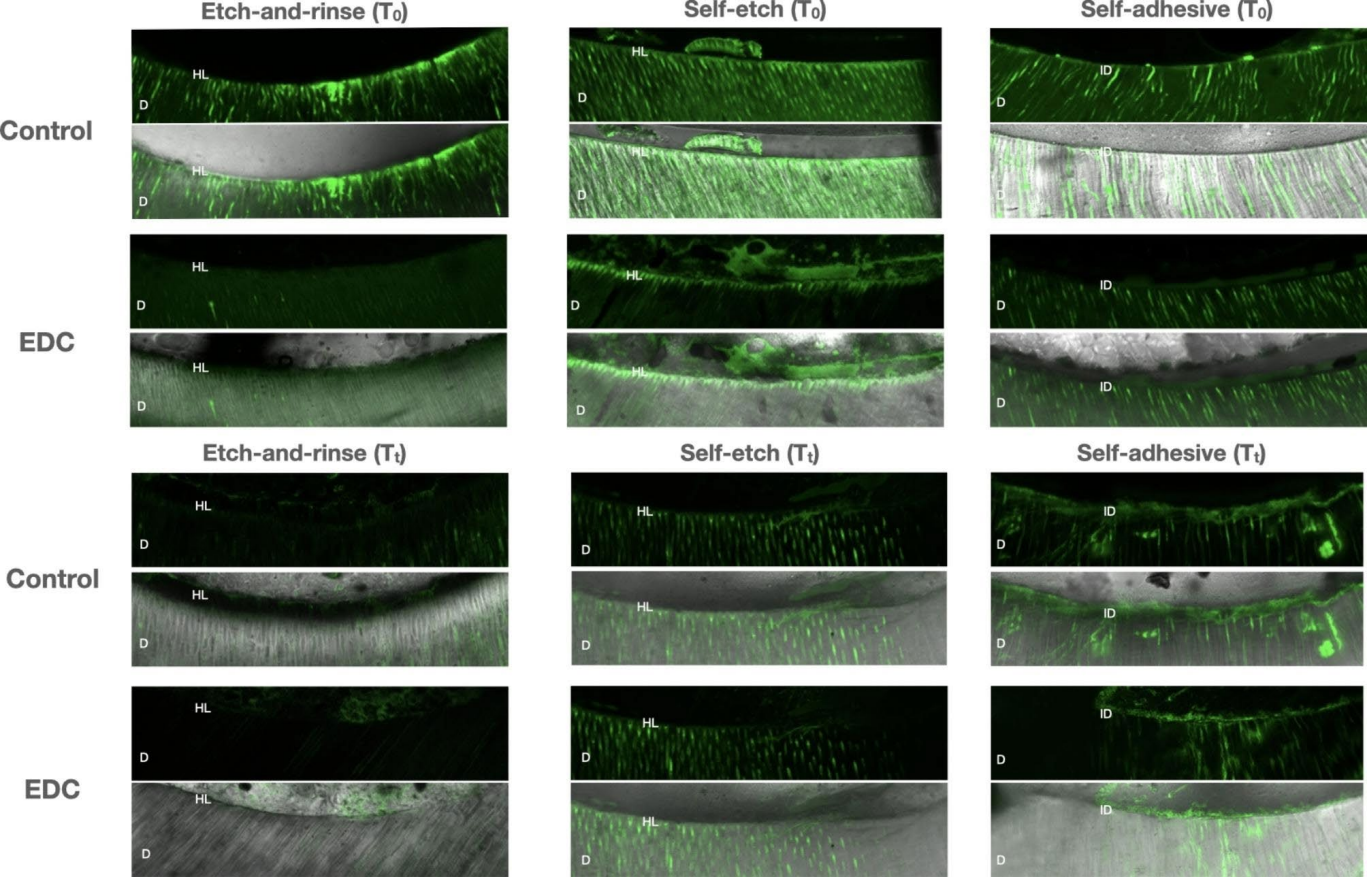
Confocal laser scanning microscopy images of resin-dentin interfaces that were incubated with quenched fluorescein-labelled gelatin. Abbreviations: D-dentin; HL-hybrid layer; ID-interdiffusion zone

Representative confocal laser scanning microscopy images of the resin-bonded radicular dentin interfaces that were incubated with quenched fluorescein-labelled gelatin are shown in Fig. 3. More prominent fluorescence, particularly within the interface with the luting agent, was observed in the control groups. Less pronounced gelatinolytic activity could be seen in the EDC groups. In general, thermocycling reduced the level of gelatinolytic activity.

### Interfacial NL expression

**Fig. 4:**
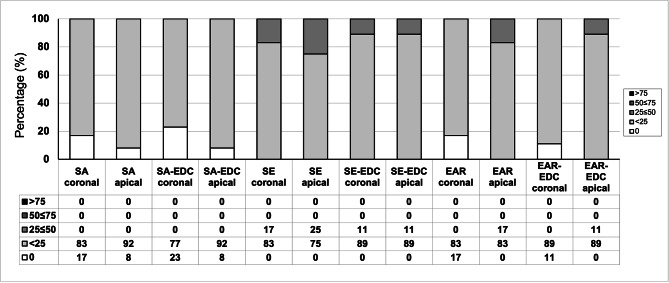
Percentage of interfacial nanoleakage expression in resin-dentine interfaces created in radicular dentine at baseline

**Fig. 5:**
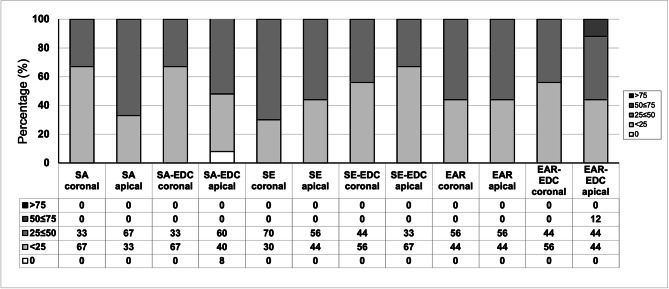
Percentage of interfacial nanoleakage expression in resin-dentine interfaces created in radicular dentine after artificial aging

The descriptive analysis of interfacial NL expression at baseline and after artificial aging is shown in Figs. 4 and 5, respectively. At baseline, SE groups seemed to be more prone to silver nitrate deposition compared to EAR and SA groups. EDC pretreatment had no influence on interfacial NL expression, and thermocycling has generally contributed to higher percentage of silver nitrate deposition along the adhesive interface in all experimental groups.

### SEM images of the fractured specimens

**Fig. 6:**
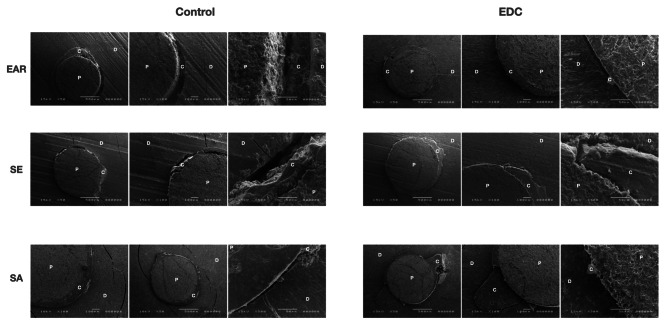
Scanning electron microscopy microphotographs (50x, 100x and 500x magnifications) of the fractured specimens at baseline. Abbreviations: D-dentin; P-post; C-cement

**Fig. 7:**
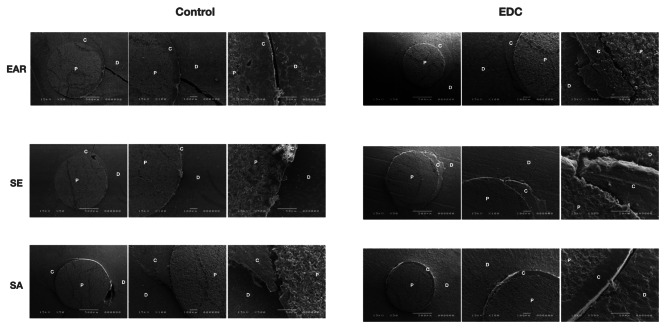
Scanning electron microscopy microphotographs (50x, 100x and 500x magnifications) of the fractured specimens after artificial aging. Abbreviations: D-dentin; P-post; C-cement

Representative SEM micrographs of the fractured specimens are presented in Figs. 6 and 7. The qualitative analysis of the SEM images showed no major differences regarding bonding interface structure between the EDC and control groups. However, the qualitative analysis of the SEM images revealed more dentin fractures and microfractures after thermocycling compared to baseline, regardless of the investigated group.

## Discussion

Debonding of fiber posts is considered the most frequent complication associated with post-retained restorations [33]. Therefore, this in vitro study aimed to investigate whether EDC application following three different cementation strategies can preserve the push-out bond strength of fiber posts, while also silencing the endogenous enzymatic activity within radicular dentin.

Root canal pretreatment with EDC had no influence on the immediate push-out strength. However, it was able to preserve the bond strength values of adhesively luted posts after artificial aging, regardless of the cementation strategy, and it reduced the level of endogenous enzymatic within radicular dentin. This led to the partial rejection of the first null hypothesis. Our results are in agreement with previous work conducted on coronal dentin, in which EDC application did not influence the immediate bond strength values [17, 18, 21]. Furthermore, the results are in line with those from Comba et al. and Shafiei et al., in which no effect of EDC on immediate push-out bond strength of fiber posts was observed when posts were cemented using a resin luting agent and an EAR adhesive system [23, 25]. The true beneficial effect of EDC pretreatment became evident only after artificial aging by means of thermocycling, as the SE and SA control groups underwent a significant reduction in bond-strength, while their respective EDC groups maintained stable values. These findings support previous research reporting a protective effect of EDC on push-out bond strength after aging up to 12 months in aqueous media when posts were luted with either SA [24] or EAR [22, 23] cementation strategy.

The ability of EDC to preserve bond strength over time when applied on acid-etched or partially demineralized coronal dentin has been previously reported [17, 18, 21], and it is considered a result of the twofold effect of EDC. Firstly, this zero-length cross-linking agent can inactivate dentin proteases in demineralized dentin matrices by altering the three-dimensional structure of MMPs and inactivating their catalytic sites. It is also able to cross-link the helical and telopeptide domains in collagen molecules and increase the stiffness of demineralized dentin matrices and HLs [5]. This study demonstrated the irrigation of a root canal with 0.3 M EDC before cementing a post with a SA resin luting agent prevented the loss of bond strength over time. Our results are in line with Lopes et al., who reported stable push-out bond strength values after aging EDC/SA-treated groups for 10 months in artificial saliva. Furthermore, they also reported that EDC contributed to the preservation of bond strength when the root dentin had been previously exposed to radiotherapy [24]. The positive effect of EDC pretreatment on the bond strength of radicular dentin is also confirmed through our failure mode analysis, which showed a lower percentage of AD fractures in EDC compared to the control groups. Regarding the interfacial nanoleakage expression, all tested groups performed similarly, but higher percentages of silver grain deposition were observed at the resin-dentin interface after artificial aging, which is in agreement with previous research [31].

A generally lower trend of endogenous enzymatic activity was observed in all EDC-treated groups compared to the control groups, both at baseline and after thermocycling. Results of the statistical analysis revealed a significant difference between EAR/EDC and SE/EDC at baseline, as well as between SA/EDC after thermocycling, which led to the partial rejection of the second null hypothesis. The etching step with phosphoric acid demineralizes dentin and activates latent MMPs with subsequent hydrolysis of the components from the hybrid layer [5]. This study demonstrated that radicular dentin pretreatment with 0.3 M EDC solution immediately after acid etching was an effective way of reducing baseline enzymatic activity, which is in accordance with previous studies [22, 23], in which 3- and 2-step EAR adhesive systems were used for luting fiber posts. Through a descriptive analysis of in situ zymography images, Alonso et al. [22] did observe generally lower enzymatic activity in EDC-treated groups after 9 months of water aging, which can be considered to be in line with our findings.

To the best of our knowledge, this is the first in situ zymography investigation on the efficacy of EDC to silence enzymatic activity when fiber posts are luted with SE and SA cementation strategies. Similarly to EAR adhesives, the application of a SE system can also activate endogenous MMPs [34]. In the SE strategy, radicular dentin was treated with a primer, after which EDC aqueous solution pretreatment was carried out, and the SE primer was then reapplied. The Multilink primer (Ivoclar Vivadent) used in this study contains phosphoric acid (data retrieved from patent literature), which can dissolve the smear layer to a certain degree and partially demineralize dentin [35], thus exposing a network of collagen fibers and MMPs, which are then deactivated by EDC. Indeed, this can be seen in the in situ zymography micrographs, in which a significantly lower level of enzymatic activity in the SE/EDC group compared to the control was found. Furthermore, considering that the pH of a freshly mixed SA resin cement can be as low as 1.5 [7], and that pH values below 5 can activate MMPs both in radicular and coronal dentin [36], it is reasonable to assume that even these simplified systems can trigger the activation of MMPs. In fact, a lower level of endogenous enzymatic activity was observed in the SA/EDC groups, especially after artificial aging, thus confirming the beneficial effect of EDC pretreatment even on non-etched radicular dentin.

Previous research reported that thermocycling reduced push-out bond strength values when fiber posts were luted with either a SE or a SA approach [28, 37]. Conversely, a decrease in bond strength values was not observed when posts were luted with the SA and EAR cementation strategies and subjected to 5.000 thermocycles [38]. These findings might be due to the study design and the low number of cycles [38] of the investigations, since 5.000 thermocycles is not enough to produce a significant effect on material properties. The results of our study correlate with previous findings [28, 37] and indicate that posts bonded with simplified cementation strategies, such as SE or SA, are more susceptible to material-tooth interface degradation, which is caused by repetitive contraction and expansion stresses during thermocycling [39].

This study investigated for the first time the influence of thermocycling on endogenous enzymatic activity within radicular dentin. When observing Fig. 1, a general trend of reduction of enzymatic activity can be observed in all groups after the samples were subjected to artificial aging. However, the only statistically significant difference was observed in the EAR/EDC and control groups. These results may seem unexpected, since the level of enzymatic activity usually increases with aging and is likely responsible for the loss of bond strength in resin-dentin interfaces [5, 21]. Although surprising, the explanation for this phenomenon might lie in the fact that, instead of aging samples in artificial saliva, as reported by Maravic et al. [21] and Breschi et al. [40], the root slices in this study were exposed to thermocycling in distilled water-filled dwells. Unlike artificial saliva, which contains zinc and calcium ions required for MMP activity, distilled water clearly represents a different storage medium. Tezvergil-Mutlua et al. investigated the requirement of zinc and calcium ions for functional MMP activity in demineralized dentin matrices. The authors reported the common use of water as an aging medium may underestimate the hydrolytic activity of endogenous dentin MMPs and can promote the loss of calcium and zinc ions from dentin matrices rather than restore them [41]. In the EAR cementation strategy, dentin demineralization was performed with phosphoric acid, and the samples were subjected to large volumes of incubation media - distilled water, which may have diluted any residues of zinc and calcium ions within demineralized dentin.

Lastly, the choice of an adequate cementation strategy which would provide long term retention to fiber posts inside the root canal can still represent a dilemma for clinicians in daily practice [42]. SA luting agents were found to be less technique-sensitive to luting procedures compared to EAR and SE cements, and they can improve the retention of posts compared to other types of luting strategies [43, 44]. The results of our study indicate all three cementation strategies performed equally well in terms of fiber post resistance to dislodgment. Similar results were observed in one of our previous studies [31], in which it was concluded that the retention of fiber posts is more material- than cementation strategy-dependent.

## Conclusion

Carbodiimide pretreatment during cementation procedures prevents the reduction of bond strength values after artificial aging, regardless of the cementation strategy employed, possibly due to the reduction of the level of endogenous enzymatic activity. Considering that pretreating radicular dentin with EDC for 1 min represents a clinically acceptable timeframe, this strategy could be of clinical interest. Furthermore, the choice of cementation strategy had no effect on fiber posts’ retention, implying that the simple and less operator-sensitive techniques can be recommended in clinical practice.

## Data Availability

The datasets used and/or analysed during the current study available from the corresponding author on reasonable request.
